# *Lawsonia intracellularis* associated equine proliferative enteropathy in Danish weanling foals

**DOI:** 10.1186/s13028-019-0447-3

**Published:** 2019-03-08

**Authors:** Anna Margareta Bohlin, Susanne Nautrup Olsen, Sigrid Hyldahl Laursen, Anna Öhman, Gaby van Galen

**Affiliations:** 1 Evidensia Equine Specialist Hospital Helsingborg, Bergavägen 3, 254 66 Helsingborg, Sweden; 2 Large Animal Teaching Hospital, Department of Veterinary Clinical Sciences, University of Copenhagen, Agrovej 8, 2630 Taastrup, Denmark; 3 Present Address: Faculty of Science, Sydney School of Veterinary Science, The University of Sydney, 410 Werombi Road, Camden, NSW Australia

**Keywords:** Diarrhea, Enteritis, Horse, Hypoalbuminemia, Hypoproteinemia

## Abstract

**Background:**

*Lawsonia intracellularis,* an obligate intracellular bacterium, causes equine proliferative enteropathy, mainly in horses around weaning. This disease is rarely reported in the Scandinavian countries.

**Results:**

Five cases of equine proliferative enteropathy were diagnosed between 2008–2016 at the University of Copenhagen Large Animal Teaching Hospital. Cases were Danish Warmbloods and a Friesian horse, aged 6–7 months, presenting with typical clinical signs of lethargy, poor body condition, pyrexia and diarrhea. Clinical pathology was consistent with previous reports of severe hypoalbuminemia and leukocytosis. Diagnosis was confirmed by fecal polymerase chain reaction, serum immunomonolayer peroxidase assay and/or immunofluorescence and fluorescence in situ hybridization performed on formalin-fixed ileum samples. Concurrent intestinal parasitism was present in all five cases. Treatment consisted of antimicrobial therapy, anti-inflammatories, intravenous crystalloids and plasma. Three foals were euthanised due to deterioration and poor response to treatment, one with complications of septic arthritis and *Strongylus vulgaris* associated intestinal infarct. The other two foals survived and were reported by the owners to be healthy on long-term follow-up.

**Conclusions:**

Equine proliferative enteropathy is a disease to consider in young horses presenting with diarrhea and hypoproteinemia in Denmark.

## Background

*Lawsonia intracellularis* causes equine proliferative enteropathy (EPE), mainly in foals around weaning [1]. The bacterium is an obligate intracellular curved Gram-negative rod [2], and infects enterocytes primarily in the ileum and distal jejunum [3, 4] causing a proliferation of the intestinal mucosal layer and subsequent malabsorption.

Typical associated clinical signs are lethargy, anorexia, pyrexia, peripheral edema, diarrhea, colic and weight loss [1, 4–6]. The most common clinicopathological findings include severe hypoalbuminemia and leukocytosis [1, 7], and abdominal ultrasound typically reveals increased small intestinal wall thickness [5, 7, 8]. Fecal polymerase chain reaction (PCR) and/or serum immunomonolayer peroxidase assay (IMPA) can be used to confirm the infection ante-mortem [1, 6, 9, 10]. Post-mortem the disease can be diagnosed by identifying *L. intracellularis* with Warthin-Starry staining (WS), immunofluorescence (IF) or fluorescence in situ hybridization (FISH) associated with proliferative lesions in the small intestine [11–15]. Treatment is by antimicrobials that achieve high intracellular concentrations [16], alongside supportive treatment with intravenous crystalloids, plasma or colloids [5].

EPE causes disease ranging from subclinical to acute and fatal [17–19]. The disease has been described as emerging in the North American horse population [20], with larger outbreaks described in the United States [8] and Canada [4]. Additionally, single cases have been reported from Switzerland [21], England [22], Belgium [23], Israel [24], Brazil [25] and Australia [26]. In Denmark, a previous seroprevalence study showed that 5% of young horses admitted to a Danish referral hospital (University of Copenhagen Large Animal Teaching Hospital) had measurable *L. intracellularis* serum antibodies [27]. However, this study was not linked to diagnosis or clinical signs. Since multiple porcine studies show a 100% specificity and no cross reactions with other pathogens for IMPA serology [28, 29], this seroprevalence study is strongly suggestive for Danish foals being exposed to the bacterium, although the test may act differently in foals compared to pigs. Reports of *L. intracellularis* infection in foals in Scandinavia is limited to a Swedish fecal PCR study on 108 clinically healthy foals 4–8 months old from four breeding farms, reporting only negative results [30] and a single short proceeding abstract where *L. intracellularis* was identified in post-mortem samples of two Danish foals [15].

Due to the lack of peer-reviewed reports of EPE in Scandinavia, the main objective of this study was to describe the clinical aspects of confirmed cases of *L. intracellularis* infection associated with EPE in a Danish referral population.

## Case presentations

Between 2008–2016 a total of five cases of EPE were diagnosed during hospitalization (n = 3) or retrospectively by IMPA on stored frozen serum samples and/or IF and FISH on formalin-fixed ileum samples (n = 2) at the University of Copenhagen Large Animal Teaching Hospital.

Cases were 6–7 months-old Danish Warmbloods and one Friesian horse, four colts and one filly, all from different premises. Cases presented in October to January in 2008 (n = 1), 2012 (n = 1), 2015 (n = 2) and 2016 (n = 1).

Mean duration of disease noted by the owner before admission was 3 days. Four cases had been treated by referring veterinarian with non-steroidal anti-inflammatory drugs (flunixin meglumine or meloxicam), and three with antimicrobials (sulfadiazine-trimethoprim or penicillin procaine) before admission. Two had received anthelmintics within 2 weeks prior to presentation (ivermectin or fenbendazole).

Confirmed cases all displayed clinical signs of lethargy, pyrexia, tachycardia and peripheral edema. A summary of clinical signs is presented in Table 1.

**Table 1:** Clinical signs, diagnostic tests, post-mortem findings and outcome in foals with equine proliferative enteropathy (n = 5)

Parameter	EPE positive (n = 5)
Clinical sign	
Lethargy	5/5 (100%)
Anorexia	5/5 (100%)
Poor body condition	4/5 (80%)
Peripheral edema	5/5 (100%)
Colic	3/5 (60%)
Diarrhea	4/5 (80%)
Pyrexia (> 38.3 °C)	5/5 (100%)
Diagnostic tests	
Intestinal parasitism^a^	
Overall	5/5 (100%)
Strongyles	4/5 (80%)
*Parascaris* spp.	1/5 (20%)
Abdominal ultrasound	
Increased small intestine wall thickness (≥ 4 mm)	4/4 (100%)
Post-mortem examination	
Small intestine	
Increased wall thickness	3/3 (100%)
Large intestine	
Colitis	2/3 (67%)
Outcome	
Survival	2/5 (40%)

*EPE* equine proliferative enteropathy

^a^Eggs, larvae or adult parasites found in feces or in the intestinal wall

All cases presented with hypoalbuminemia, hyponatremia, hypomagnesemia, hypocalcemia, increased fibrinogen and variably increased serum amyloid A, as shown in Table 2. Hypoalbuminemia became more profound during hospitalization for all cases (10.0–15.0 g/L). Four cases presented initially with normal leukocyte counts, however, three developed leukocytosis during the course of disease (17.0–28.5 × 10^9^ cells/L).

**Table 2:** History, clinical signs, laboratory values (at admission) and clinical pathology of foals with equine proliferative enteropathy (n = 5)

Parameter	Unit	Reference	Mean ± SD	Range	Number
History					
Duration of disease before admission (days)		–	3 ± 2	1 to 7	5
Clinical signs					
Heart rate	Bpm	28–44	*58 ± 9*	*48 to 76*	5
Respiratory rate	Rpm	12–24	18 ± 5	12 to 24	5
Complete blood count and biochemistry					
Packed cell volume	L/L	0.32–0.50	0.41 ± 0.07	0.37 to *0.52*	5
Leukocyte count	10^9^/L	5.45–12.65	11.51 ± 3.14	8.47 to *17.00*	5
Neutrophil count	10^9^/L	2.26–7.22	6.13 ± 3.47	*1.20 to 10.80*	5
Serum protein	g/L	57.0–74.0.	*30.1 ± 4.2*	*25.1 to 34.6*	5
Albumin	g/L	28.0–40.0	*15.8 ± 2.8*	*13.0 to 19.7*	5
Fibrinogen	g/L	0.0–4.0	*6.3 ± 2.2*	*4.2 to 10.6*	5
Serum amyloid A	mg/L	0–30	*943 ± 1366*	*108 to 3643*	5
Iron	μmol/L	13.1–43.0	*10.0 ± 3.4*	*4.0 to 12.8*	5
Gamma-glutamyl transferase	U/L	4–19	6 ± 2	4 to 8	4
Total bilirubin	μmol/L	0.0–52.0	34.7 ± 8.2	22.7 to 41.9	5
Creatinine	μmol/L	30–130	102 ± 18	80 to 129	5
Creatinine kinase	U/L	0–348	267 ± 127	134 to *487*	5
Asparate transaminase	U/L	228–366	*168 ± 38*	*128 to 227*	5
Sodium	mmol/L	136–142	*122 ± 3*	*119 to 126*	5
Potassium	mmol/L	2.6–5.0	3.1 ± 0.4	2.8 to 3.8	5
Chloride	mmol/L	99–109	*98 ± 4*	*93* to 105	5
Ionised calcium	mmol/L	1.40–1.72	*1.26 ± 0.08*	*1.14 to 1.34*	5
Total magnesium	mmol/L	0.66–0.95	*0.43 ± 0.08*	*0.29 to 0.52*	5
Lactate	mmol/L	0.0–2.0	*2.8 ± 2.0*	0.7 to *6.2*	5
Glucose	mmol/L	4.3–9.0	7.8 ± 2.4	6.5 to *11.3*	5
pH		7.32–7.55	7.32 ± 0.07	7.20 to 7.40	5
Bicarbonate	mmol/L	24.0–30.0	*20.0 ± 1.5*	*18.0 to 22.0*	4
Base excess		− 4.0–4.0	*− 6.1 ± 0.9*	*− 7.1 to − 6.2*	3
Triglycerides	mmol/L	0.1–0.9	0.7 ± 0.1	0.6 to 0.8	2
Peritoneal fluid					
Protein	g/L	0.0–10.0	4.5 ± 7.8	0.0 to *18.0*	4
Total nucleated cell count	10^9^/L	0.0–10.0	5.3 ± 6.2	0.2 to *14.2*	3

Italics text: value outside reference range

Three cases had feces sampled for *L*. *intracellularis* real time PCR (Laboklin Gmbh & Co, Bad Kissingen, Germany) within the first 48 h of admission, confirming EPE. For the other two cases testing for *L*. *intracellularis* was not performed during hospitalization. A summary of diagnostic tests performed for included cases is presented in Table 3.

**Table 3:** Diagnostic test results of foals diagnosed with equine proliferative enteropathy 2008–2016

Case	Age (months)	Outcome	Fecal rtPCR	IMPA (admission)	IMPA (8 weeks)	WS^a^	IF^a^	FISH^a^
1	7	NS	*Positive*	*1:100*	NP	Negative	Negative	Negative
2	6	S	*Positive*	< 1:25	NP	NP	NP	NP
3	6	S	*Positive*	1:50	*1:200*	NP	NP	NP
4	7	NS	NP	*1:480*	NP	Negative	*Positive*	Negative
5	6	NS	NP	< 1:25	NP	NP	*Positive*	*Positive*

Italics: confirmative of equine proliferative enteropathy

*NS* non-survival, *S* survival, *rtPCR* real time polymerase chain reaction, *IMPA* immunomonolayer peroxidase assay, *WS* Warthin-Starry staining, *IF* immunofluorescence, *FISH* fluorescence in situ hybridization, *NP* not performed

^a^Formalin-fixed ileum samples

**Fig. 1:**
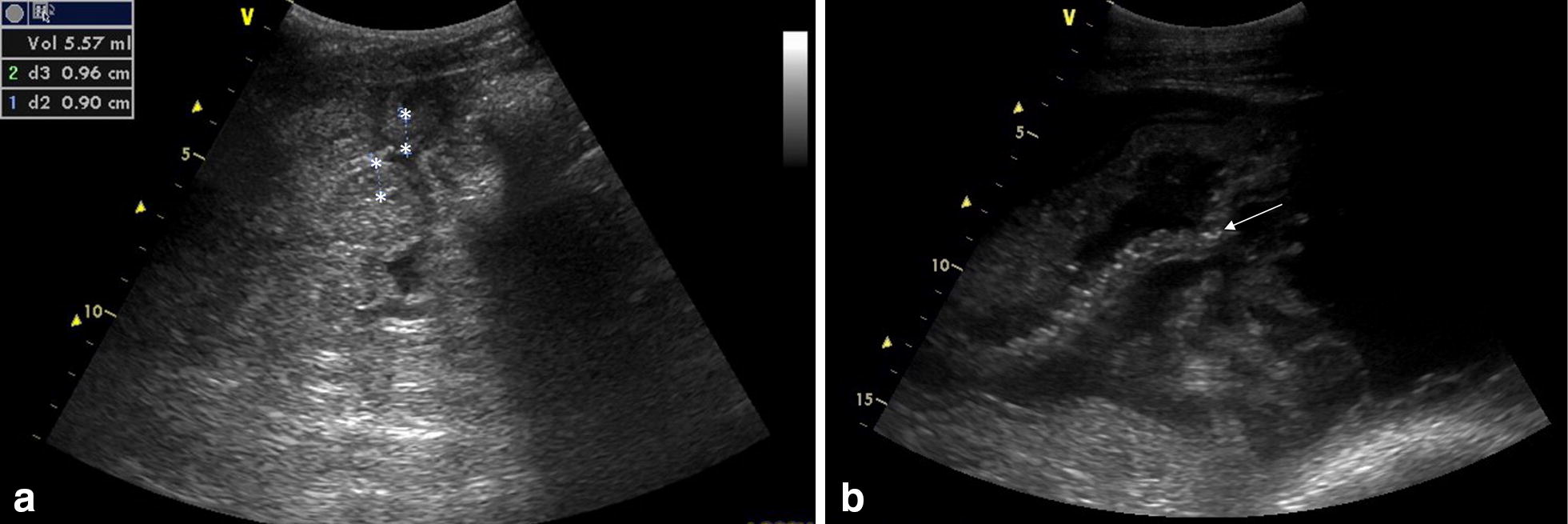
Ultrasound images obtained in a foal with confirmed *Lawsonia intracellularis* infection. **a** Cross sectional view of small intestines with increased wall thickness of 0.9 cm (asterisk). **b** Longitudinal view of a small intestinal wall with increased echogenicity and thickness (arrow)

Concurrent intestinal parasitism was present in all five cases with either strongyles (240–300 eggs per gram (EPG) or grossly present on necropsy) or *Parascaris* spp. (340 EPG). Three cases (case 1, 2 and 4) had a fecal culture for *Salmonella* spp. performed and one (case 2) had *Clostridium difficile* toxin A and B and *Clostridium perfringens* enterotoxin analyzed, but all were found negative. Peritoneal fluid was analyzed for four cases (case 1–3 and 5). The sample was mildly inflammatory in case 5, for the others samples were within normal limits. Abdominal ultrasound (Fig. 1) was performed in four cases (case 1–3 and 5) and showed increased small intestinal wall thickness for all (0.5–0.9 cm; normal range 0.2–0.3 [31]).

All five cases were treated with nonsteroidal anti-inflammatories (flunixin meglumine^1^ or meloxicam^2^), crystalloids (Ringer’s-acetate^3^or sodium chloride 0.9%^4^) and colloids (hetastarch 6%^5^ and equine plasma^6^). Partial parenteral nutrition was provided with glucose 50%^7^ or amino acids 11.4%^8^ for three foals (case 1–3). One surviving foal (case 3) initially received benzyl penicillin^9^ and gentamicin^10^ but was changed to rifampin^11^ and clarithromycin^12^ when EPE was suspected on day 2. Due to poor response, the foal received oxytetracycline^13^ from day 4. The other surviving foal (case 2) was treated with oxytetracycline (see Footnote 13). One non-surviving foal received erythromycin^14^ and rifampin (see Footnote 11) (case 1), one received benzyl penicillin and metronidazole (case 5) and the last non-survivor did not receive antimicrobial treatment (case 4). Additionally, sucralfate^15^ omeprazole^16^ and oral electrolytes were given to four cases. Three cases showed signs of colic at several occasions and were administered metamizole^17^ butorphanol^18^ and butylscopolamine^19^ Additionally, treatment with dexamethasone^20^ enoxaparin^21^ glutamin^22^, neostigmine^23^ calcium gluconate^24^ lidocain^25^ and bicarbonate was provided. Three foals were given anthelmintics during hospital stay (moxidectin^26^ and fenbendazole^27^) and one foal received anthelmintic treatment after discharge.

Three foals (case 1, 4 and 5) were euthanised after 4–5 days of treatment, due to sudden deterioration and poor prognosis. Post-mortem examination of case 4 showed severe generalized edema and grossly increased small intestinal wall thickness. Histopathology of the duodenum and jejunum showed muscular hypertrophy, and in the ileum a few mineralized foci and a crypt abscess was found, however samples had autolytic changes and could not be fully evaluated. On retrospective analysis of serum, the foal had an IMPA titer of 480 (normal range: < 60 [1, 6]) (National Veterinary Laboratory, Technical University of Denmark (NVL)) and was positive by IF on formalin-fixed samples of the ileum (NVL).

Gross pathology of case 5 showed cachexia, hypertrophy of the small intestinal mucosa and multiple encysted cyathostomins in the colon wall. Histopathology of small intestinal sections were unremarkable, but formalin-fixed samples from the ileum were positive for *L. intracellularis* by IF and FISH.

Case 1 presented with additional symptoms to the classical signs of EPE, with a respiratory noise from moderate airway edema at admission. This foal was diagnosed with EPE based on a positive fecal PCR and a serum IMPA titer of 100. On day 3 of hospitalization, this foal developed septic arthritis of the tarsocrural joint. On the 4th day of treatment the foal’s general condition rapidly worsened with circulatory compromise and development of severe pulmonary edema. Due to poor response to treatment, the foal was euthanised. Post-mortem examination showed gross generalized edema and increased small intestinal wall thickness. Additionally, the foal had peritonitis and an arterial thrombus causing infarction of the pelvic flexure of the colon. In the ileocolic artery, there was evidence of chronic arteritis with aneurysms and thrombi containing *Strongylus vulgaris* larvae. Histopathology showed generalized thickened small intestine with chronic ulcerative enteritis and lymphocytic hyperplasia. Testing for *L. intracellularis* by IF and FISH was negative for this case.

The two surviving foals (case 2 and 3) were hospitalized for 5 and 11 days, respectively. Case 3 had a re-visit 8 weeks after discharge where it was found clinically normal except for poor body condition and mild exfoliative dermatitis of the cannon bones and ear tips. Abdominal ultrasound was normal, complete blood count and biochemistry showed normal albumin (28.9 g/L) and leukocytes (10.1 × 10^9^ cells/L), and mildly increased fibrinogen level (5.1 g/L). Serology at this time point revealed an IMPA titer of 200 compared to the admission titer of 50 (Laboklin Gmbh & Co). On follow-up 1 year later the owner reported the colt to be healthy with a normal body condition. Case 3 had follow-up examination 4 days after discharge, where ultrasound showed small intestinal thickening to have decreased, and serum albumin to be low but stable (15.0 g/L). Approximately 2 years after discharge the owner was contacted by telephone and reported the colt to be healthy.

Comparing blood samples between surviving and non-surviving cases, these were not different regarding severity of leukocytosis, hypoproteinemia, hypoalbuminemia, fibrinogen or serum amyloid A values.

## Discussion and conclusions

To the authors’ knowledge, this is the first peer-reviewed report describing the clinical aspects of foals with *L. intracellularis* associated EPE in Scandinavia. This study supports that *L. intracellularis* not only circulates and foals are in contact with the bacterium, but also causes clinical disease (EPE) in Denmark. Therefore, EPE should be considered a differential diagnosis in Danish weanlings.

*Lawsonia intracellularis* is present in the majority (94%) of Danish pig farms [32]. A screening for *L. intracellularis* was performed on stored serum samples at the Large Animal Teaching Hospital, University of Copenhagen from horses admitted 2005–2008, regardless of health status and/or diagnosis. Five percent of horses between 0 days and 24 months of age had detectable antibodies by IMPA [27]. It has been shown in experimental studies that porcine strains can cause disease in horses [13], however, equine strains cause more severe disease [33], and is genetically slightly different from the porcine strains [34]. In the confirmed EPE cases of this report, there was no known association with pigs, and no strain assessment was performed.

Compared to reports from the USA and Canada [4, 8], only few cases were found in this study and none of them occurred in outbreaks. Yet, the cases presented were all within the expected age group of around 6 months, and presented at the time of year when EPE is most frequently reported in other studies (September-January) [1]. Confirmed cases showed the typical clinical signs associated with EPE with lethargy, anorexia, pyrexia, peripheral edema, colic and diarrhea [1, 17], and the typical clinicopathological findings of hypoalbuminemia and leukocytosis [1]. For cases where abdominal ultrasound was performed, marked thickening of the small intestinal wall was seen. This is reported a common finding in EPE cases, with reports of wall thickness of up to 1.0 cm [5, 7, 8, 35]. However, in some cases abdominal ultrasound is normal despite confirmed EPE, whereby this technique cannot be used to rule out the disease [1, 17]. Abdominal ultrasound is an important diagnostic tool and should be performed in cases suspected of EPE.

Fecal real time PCR was used to diagnose EPE in three of the reported cases; the other cases described in the current study did not have fecal samples submitted. The fecal PCR assays have been evaluated in pigs, showing very high specificity in several studies (100%), whereby false positives are unlikely [36–38]. The reported sensitivity is lower, ranging from 71% (early), and 38% (later) in the course of disease [11]. In studies of equine weanlings, including only cases with positive serology and clinical signs suggestive of EPE, 74–79% of fecal samples were positive on PCR [1, 6]. There is a greater chance of positive fecal PCR if cases are sampled early in the course of disease, and before treatment with antimicrobials [1]. Our positive cases were sampled the day after arrival and had only received trimethoprim-sulfadiazine and penicillin procaine, i.e. antimicrobials without intracellular penetration.

An additional ante-mortem diagnostic method is serology by IMPA. In this study two different laboratories performed the IMPA analyses, with results reported in different dilutions of 50 or 60. In previous studies, foals have been considered seroconverted when IMPA titers were ≥ 60 [1, 6], and higher titers have correlated with more severe disease [17]. Recently also an enzyme-linked immunosorbent assay test was validated for the detection of *L. intracellularis* antibodies in horses [9]. Serology in the current study was confirmative of EPE for three cases (titers 100–480). In one of these, the titer was below 60 on admission, but was significantly increased when re-measured 8 weeks later (50 compared to 200). The largest study on EPE published had 80% of cases positive by IMPA, and explained negative results most likely being a result of too early sampling [1]. In an experimental study, antibodies by IMPA in weanling’s after challenge with *L. intracellularis* was first detected after 2–3 weeks [17]. This could potentially explain the low titers in two cases positive by real time PCR or IF and FISH (case 2 and 5), with short duration of disease before admission, where detectable antibodies may not yet have been produced.

In this study, three techniques for post-mortem identification of *L. intracellularis* on formalin-fixed ileum samples were used. Warthin-Starry staining yielded negative results in the three non-surviving cases that were positive by other ante-mortem (real time PCR or IMPA) or post-mortem tests (IF and FISH). All post-mortem tests rely on the presence of bacteria or bacterial antigen in the tissue, with IF and FISH considered more sensitive and specific compared to WS [11, 12]. Previous antimicrobial treatment could have affected the results, since it has been reported that infected foals stop shedding the organism within 4 days after treatment is initiated [39]. The one foal positive by fecal real time PCR and serum IMPA (case 1) but negative on post-mortem tests, had received antimicrobial treatment (erythromycin and rifampin) with a duration of 4 days before euthanasia, possibly explaining these negative post-mortem findings.

Diagnosis of EPE is furthermore complicated by concomitant diseases with similar clinical presentation that frequently occur in foals, especially intestinal parasitism. This is well described in the literature [1, 3–5, 7, 8], and is also a finding in the current study. This can account for underdiagnosing of EPE. Concurrent intestinal parasitism with strongyles or *Parascaris* spp. was found in all cases, making parasitism one of the major differential diagnoses of EPE. It should be stressed that in the case of cyathostomiasis in foals within the age group 4–8 months, additional testing for EPE should be performed. It may be speculated that concurrent intestinal parasitism could make the foal more susceptible to *L. intracellularis* infection. In one non-surviving EPE case in the current study, chronic arteritis of the ileocolic artery and thrombi associated with *S. vulgaris* was diagnosed at necropsy. This was likely an important contributing factor to the deterioration and subsequent euthanasia of this foal. In the literature, there is evidence of coagulation disturbances in the form of epistaxis and hematochezia [19] and infarction from arterial thrombi [1, 3, 8] associated with EPE. This has been reported in severely affected cases with suspicion of translocation of bacteria over the inflamed and infected intestinal mucosa, and subsequent sepsis [19]. In these cases, the formation of thrombi has been attributed to severe hypoproteinemia and depletion of coagulation factors, along with severe systemic inflammatory response syndrome (SIRS) due to sepsis. Our case had evidence of necrotizing enteritis on post-mortem examination, and evidence of sepsis from a septic joint. To our knowledge, this is the first case presented with confirmed EPE and concurrent *S. vulgaris* associated thromboembolism. However, it cannot be completely ruled out that thrombi and deterioration was also caused by sepsis and SIRS.

Based on the severity of hematologic and biochemistry changes, it was not possible to distinguish survivor from non-survivors in the current case series. Survival was only 40% in the current study, a low number in comparison to the 93% previously reported in a study of 57 EPE affected foals from the USA [1]. Survival may have been affected by the advanced state of disease on admission, the choice of antimicrobials (both surviving foals were treated with oxytetracycline, and non-survivors instead received penicillin, metronidazole, erythromycin, rifampin or no antimicrobials), or the coinciding pathologies. Unfortunately, the low number of included foals prevented statistical analysis of risk factors. However, long term prognosis for survivals seemed favorable, with the two surviving foals reported to be healthy on follow-up 1 and 2 years later.

Limitations of this study are mainly due to the partially retrospective nature, including lack of some clinical and laboratory data and inconsistent diagnostic testing. Testing all suspected cases by fecal PCR early in disease, and later by serum IMPA, would have been preferable to establish diagnosis and optimize treatment.

In Denmark, *L. intracellularis* was confirmed to cause clinical disease (EPE) in five weanling foals. Concurrent intestinal parasitism, an important differential diagnosis, was present in all foals. Therefore, the possibility of EPE should be considered in foals that are presented with diarrhea, pyrexia and/or severe hypoalbuminemia in this country, even if a diagnosis of intestinal parasitism has been made already. Presentation was similar as in other parts of the world, with good response of the survivors in long term.

## Abbreviations

EPE: equine proliferative enteritis
FISH: fluorescence in situ hybridization
IF: immunofluorescence
IMPA: immunomonolayer peroxidase assay
NVL: National Veterinary Laboratory, Technical University of Denmark
PCR: polymerase chain reaction
SIRS: severe inflammatory response syndrome
WS: Warthin-Starry staining

## Footnotes

1 Findyne 50 mg/mL, Intervet.

2 Metacam 20 mg/mL, Boehringer Ingelheim Vetmedica.

3 Ringer-acetat Fresenius Kabi, Fresenius Kabi.

4 Natriumklorid 9 mg/mL, Fresenius Kabi.

5 Voluven 60 mg/mL, Fresenius Kabi.

6 Hypermune Equine Plasma, Veterinary Immunogenics.

7 Glucos 500 mg/mL, Fresenius Kabi.

8 Vamin 18 g N/I elektrolytfri, Fresenius Kabi.

9 Benzyl penicillin 1.2 g, Panpharma.

10 Genta-Equine 100 mg/mL, Dechra.

11 Rimactan 300 mg, Sandoz.

12 Clarithromycin 500 mg, Orion Pharma.

13 Engemycin 100 mg/mL, Intervet.

14 Hexabotin 500 mg, Durascan.

15 Antepsin 1 g, Orion Pharma.

16 GastroGuard 370 mg/g, Merial.

17 Vetalgin 500 mg/mL, Intervet.

18 Torbugesic 10 mg/mL, Orion Pharma.

19 Buscopan 20 mg/mL, Boehringer Ingelheim.

20 Dexadreson 2 mg/mL, Intervet.

21 Klexane 100 mg/mL, Sanofi.

22 l-Glutamine, Scientec Nutrition.

23 Neostigmin 2.5 mg/mL, SAD.

24 Calciject 40, Norbrook.

25 Lidocain 20 mg/mL, Mylan.

26 Equest 18.9%, Orion Pharma Animal Health.

27 Panacur 18.5%, MSD Animal Health.
